# Key factors influencing orthopaedic operating room contamination: Impact of human activity and *Staphylococcus epidermidis* prevalence

**DOI:** 10.1002/jeo2.70321

**Published:** 2025-07-18

**Authors:** Nicolas C. I. Ion, Sorin R. Fleaca, Bogdan‐Axente Bocea, Cosmin‐Ioan Mohor, Mihai‐Dan Roman, Alexandru‐Florin Diconi, Adrian N. Cristian, Doru‐Florian‐Cornel Moga, Mona Y. Cataniciu, Ovidiu N. Popa, Adrian G. Boicean, Victoria Birlutiu

**Affiliations:** ^1^ Faculty of Medicine Lucian Blaga University of Sibiu Sibiu Romania; ^2^ County Clinical Emergency Hospital Sibiu Romania; ^3^ Clinical Military Emergency Hospital Sibiu Romania

**Keywords:** hip, knee, operating room, orthopaedic, Staphylococcus

## Abstract

**Purpose:**

The presence of pathogen microorganisms in the operating room remains a significant concern. This study aims to explore the factors influencing the presence of pathogens in the operating room.

**Methods:**

We analysed the presence of microorganisms in the operation theatre during total knee and hip replacement procedures for 33 patients performed in the same operation room (OR) over a period of 2 months. Three hundred and ninety‐six samples were taken from several areas belonging to the sterile field. We have also analysed behavioural aspects in the OR that could influenced the potential contamination of the sterile field, such as the number of touches of the lamps, the temperature in the OR, the height of the operating table, the number of door openings.

**Results:**

Out of the total of 396 samples, 74 were positive. Most contaminations were with *Staphylococcus epidermidis* (45.45%). Most pathogens were found on the instrument table at the end of the intervention (27.27%). There is a correlation regarding the fact that at lower heights, the risk of contamination higher than if the table is raised more. We also find correlations between the number of operating room doors opened and the presence of field contamination, as well as between the number of touches of the lamps and the presence of pathogens on sterile fields.

**Conclusion:**

The presence of pathogens in the OR is influenced by the number of lamp touches, the frequency of door openings, and the increased number of people in the room. However, temperature and the height of the operating table do not have a significant impact on the occurrence of pathogens. The most commonly found pathogen in the OR was *S. epidermidis*. At the end of the surgery, the instrument table showed the highest percentage of pathogen presence.

**Level of Evidence:**

Level IV, case series.

**Abbreviations**
ASA: American Society of Anesthesiologists
CBC: complete blood count
OR: operating room
SPSS: statistical product and service solutions
SSI: surgical site infections
SUCH: Sibiu University Clinical Hospital

## INTRODUCTION

The operating room is a critical environment where surgical interventions are performed with the primary goal of improving patient health. However, despite accurate infection control measures, the presence of pathogens in the operating room remains a significant concern. Pathogens are microorganisms capable of causing infections, and their introduction into the operating room can lead to postoperative complications [9] and also periprosthetic infections, especially that the number of osteoarthritis is increasing every year [2, 3, 8]. There are instances when the very environments designed to promote health can harbour hidden dangers. The sterile surfaces of operating rooms are not exempt from this risk. The discovery of pathogens within the orthopaedic operating room of the Sibiu University Clinical Hospital (SUCH), has raised concerns and shed light on the importance of maintaining the highest standards of hygiene and infection control practices. Several risk factors have been discovered to increase the postoperative early infection rate [27].

Ensayef et al., published in 2009 and involving 1216 swabs collected from different surfaces in the operating theatre, they have found a rate of positive cultures of 3.7% [6]. Orthopaedic procedures require aseptic environments to minimise the risk of infections especially that most of the advanced aged patients suffer from rheumatoid arthritis, an American Society of Anesthesiologists (ASA) risk score >2, diabetes mellitus, immune‐compromission, morbid obesity or previous revision arthroplasty [4, 10, 12]. The recent identification of pathogens within the sterile surfaces of the operating room has raised questions about the potential consequences for patients and the protocols in place to protect them and for sure, airborne contamination control is also very important requirement in operating theatres environments to assure patient safety and medical staff health [23].

Pokrywka et al. affirmed in his study that an increase in the bacterial counts of airborne microorganisms is noted during increased activity levels within the OR including a high number of door openings [21].

In 2012, Laham et al. published an article where he mentioned that from a total of 243 swabs investigated, 24.7% were contaminated and the highest prevalence of bacteria was represented by *Staphylococcus* spp. (45.3%) [1].

Factors such as increased human activity, frequent door openings, and equipment handling could contribute to intraoperative contamination. In this article, we aim to determine whether the factors studied pose a risk for intraoperative contamination and to determine the presence or absence of pathogens in areas typically considered sterile.

## MATERIALS AND METHODS

Our study concentrated on total knee and hip arthroplasty surgeries in 33 chronic patients admitted to the SUCH. Both cemented and uncemented prostheses have been used in total arthroplasty procedures.

We recorded the start and end time of each procedure. We have analysed only first procedures performed in the same OR. We also monitored the temperature in the operating room during procedures, which varied between 18°C and 22°C.

Another analysed variation with potential implication in contamination was the number of medical personnel in the OR.

We also measured the height of the operating table used during the interventions, with values between 100 and 150 cm. We recorded the number of operating theatre door openings, as well as the number of lamp taps.

To assess the presence of pathogens in the operating room, we collected samples in tubes with AmiesViscosa transport media at three different time points: at the beginning of surgery, 60 min after the start of surgery, and at the end of surgery. Samples were taken from sterile surfaces, including the sterile drape in the incision area, at a distance of 10 cm from the incision on the sterile isolation field (Hip/Knee Artrhroplasty Surgery Pack from Submed) and at a distance of 50 cm from the same sterile field. We also collected samples from the handle of the lighting lamps and from the instrument table.

The study was conducted for patients operated on in the same operating room, with an area of 47.09 square metres, with a volume of 126 cube metres.

Statistical analysis was performed with SPSS software package. The level of significance was set at *p* ≤ 0.05, indicating statistical significance for all analyses.

## RESULTS

Eighteen patients underwent knee arthroplasty, while the remaining 15 underwent hip arthroplasty. At the level of the handles of the lighting lamps, no pathogen was identified during the entire surgical intervention. Between 0 and 5 touches the percentage of bacteria appeared was 40%, between 5 and 10 it dropped to 30% and between 10 and 15 touches it remained the same. There is also a correlation between the number of people in the operating room during surgery and the number of new pathogens. Therefore, for nine people being in the OR, the percentage of new germs identified was the highest (45.45%). At seven and eight people present, there was a 9.09% increase in germs, and at 10 and 11 an increase of 18.18%.

The significant decrease in bacterial presence between 0–5 and 5–10 touches suggests that initial contacts contribute the most to contamination. After five touches, bacterial percentages level off, as evidenced by the non‐significant difference between the 10–15 and 5–10 touch groups (Table 1).

**Table 1:** Pairwise comparisons using Tukey HSD post hoc test of pathogen frequencies across lamp touches.

Group 1	Group 2	Meandiff	*p*‐adj	Lower	Upper	Reject
0–5 Touches	10–15 Touches	−11.0418	0	−16.1249	−5.9587	True
0–5 Touches	5–10 Touches	−9.3044	0.0003	−14.3875	−4.2213	True
10–15 Touches	5–10 Touches	1.7374	0.6773	−3.3457	6.8205	False

Abbreviation: HSD, honestly significant difference.

**Figure 1:**
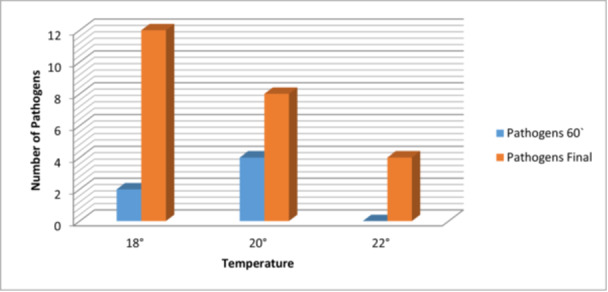
The correlation between new pathogens in the operating room and temperature at different operation stages.

At a temperature of 18°C, we can observe an additional presence of pathogenic germs, at 60 min, compared to the beginning of the operation, so we were able to observe the appearance of another two pathogens on the sterile fields. At the end of the operation another 12 pathogens were identified. At a temperature of 20°C, the number of pathogens present increases by another four at one hour after the start of the operation and by another eight at its end. At 22°C, the number of pathogens identified did not change after the first our of surgery, but increases by 4 at its end (Figure 1). The association between temperature and pathogen counts in this dataset is n.s. (Table 2).

**Table 2:** Pairwise comparisons using Tukey HSD post‐hoc test of pathogen frequencies across temperature variations in OR.

Group 1	Group 2	Meandiff	*p*‐adj	Lower	Upper	Reject
18°C	20°C	−1	0.9755	−20.5999	18.5999	False
18°C	22°C	−5	0.5932	−24.5999	14.5999	False
20°C	22°C	−4	0.7014	−23.5999	15.5999	False

Abbreviations: HSD, honestly significant difference; OR, operation room.

**Figure 2:**
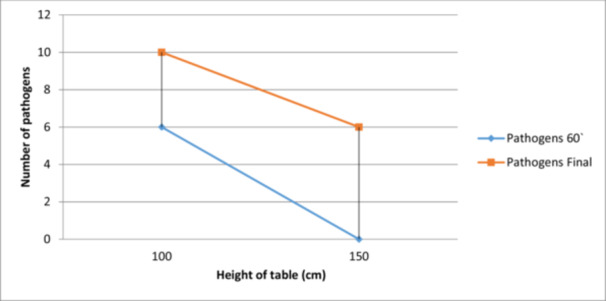
The correlation between surgical table height during surgery and new pathogens at different operation stages.

During the study, it was possible to observe an increase in the number of pathogens present at 60 min from start, as well as at the end of the intervention for a table height of 100 cm. Therefore, for the table height of 100 cm, it was possible to observe the appearance of six new pathogens one hour after the beginning of the operation and another 10 at the end of the operation, while at 150 cm height, no other pathogens were identified at 60 min, but six new pathogens could be identified at the end of the surgery (Figure 2). The association between the height of the table and the pathogen presence is n.s. (Table 3).

**Table 3:** Pairwise comparisons using Tukey HSD post‐hoc test of pathogen frequencies across operating table height during surgery.

Group 1	Group 2	Meandiff	*p*‐adj	Lower	Upper	Reject
100 cm	150 cm	−5	0.2999	−20.5134	10.5134	False

Abbreviation: HSD, honestly significant difference.

**Figure 3:**
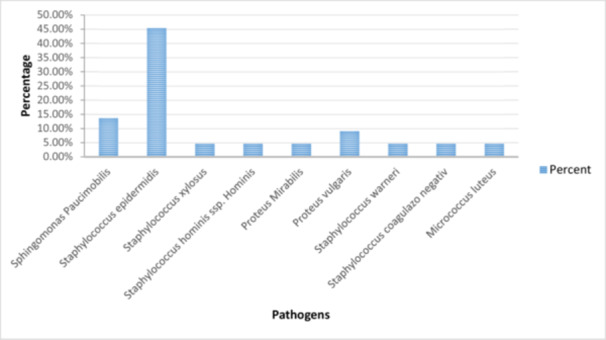
The percentage of pathogens identified in the operating room during surgical interventions.

There is a significant difference between the observed and expected frequencies of the pathogens. During the 33 interventions, we collected 396 samples that were sent to be analysed. Of the total samples collected, 74 of them were positive for several types of pathogens. The majority of germs were represented by *Staphylococcus epidermidis* (coagulase‐negative, gram‐positive cocci bacteria) [16], which was 45.45% of the total. Sphingomonas Paucimobilis (gram‐negative bacillus) [11] is in second place, with a percentage of 18.31%. Proteus vulgaris represents a percentage of 9% of the total germs present. Other pathogens identified in the sterile fields but in smaller percentages were: *Staphylococcus xylosus* (commensal bacterium) [5], *Staphylococcus hominisssp* (gram‐positive, coagulase‐negative bacteria) [26], *Proteus mirabilis* (gram‐negative facultative anaerobe) [14], *Staphylococcus warneri*, *Micrococcus luteus* (catalase‐, oxidase‐, gram‐positive cocci) [29]. The percentage of those listed above is 4.54% of the total number of pathogens identified (Figure 3). One positive thing is that other studies showed that *Staphylococcus aureus* is one of the common infecting organisms after periprosthetic joint surgery [18] and it represents a frequent intra‐ and extracellular pathogen associated with orthopaedic infections [22], but in our study, we did not indentify it.

Most of the germs encountered were identified on the instrument table at the beginning of the operation, being approximately 17.18%. The number increased to approximately 26.27% at the end of the intervention, and at one hour from the beginning of the intervention it was 8%. At the level of the incision, the percentage of pathogens present at the beginning of the intervention was 8%, it was maintained one hour after the beginning of the operation, but increased at the end to 17.18%. On the sterile field, 10 cm away from the incision site, a percentage of 8% could be observed, which was maintained throughout the intervention period. The same thing happened 50 cm from the incision site, with a percentage of 3.54% (Table 4).

**Table 4:** The percentage of identified pathogens during surgery based on sampling area and time of collection.

	0′ (%)	60′ (%)	Final (%)
Incision	8	8	17.18
Sterile field 10 cm	8	8	8
Sterile field 50 cm	3.54	3.54	3.54
Lamp	0	0	0
Instrumenting table	17.18	17.18	26.27

**Figure 4:**
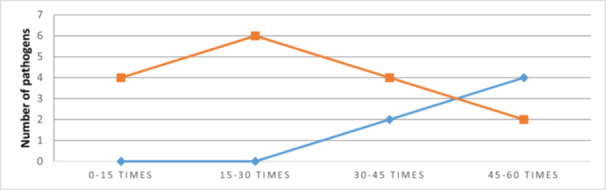
The correlation between door openings during surgery and new pathogens at 60 min and completion.

There is a significant difference between the observed and expected frequencies., The distribution of pathogens is significantly associated with the number of door openings. It increases in direct proportion the length of the case and also have an exponential relationship with the number of persons being in the operating room [17]. Entering and exiting the OR, or create unnecesary traffic and movement during the intervention, the intended airflow around the open wound becomes disrupted and does not remove the airborne contaminants [21]. Therefore, a correlation can be observed between the number of openings and the number of new pathogens. 6 was the maximum number of new sprouts at the end of the operation and it was reached between 15 and 30 door openings. The number of door openings between 0 and 15 times influenced the presence of germs. They increased by four at the end of the operation. Between 30 and 45 openings, the number of pathogens increased by two at 60 minutes from the start of the operation and by another four at the end of the intervention. Between 45 and 60 openings, the number increased by four at one hour from the beginning and by another two pathogens at the end of the operation (Figure 4).

The highest concentration of pathogens, 40%, was found at 0–5 lamp touches. Between 5 and 10 lamb touches, the pathogen presence decreased to 30%, and this same level was observed at 10–15 touches of the lamp. There is a significant difference between the observed and expected frequencies. The distribution of pathogens is significantly associated with the number of lamp touches.

When there were seven or eight persons in the OR, the pathogen percentage was relatively low at 9.09%. However, with nine persons present, the percentage of pathogens significantly increased to 45.45%. As the number of persons increased to 10 or 11, the pathogen levels decreased to 18.18%, indicating a fluctuating but notable presence of pathogens based on the number of individuals in the OR. There is a significant difference between the observed and expected frequencies. Therefore, the distribution of pathogens is significantly associated with the number of persons in the operating room.

## DISCUSSION

The presence of pathogenic germs on sterile fields in the operating room and during surgery, along with the determination of the antibiogram of each pathogen and the influence of antibiotics in the prophylaxis of periprosthetic infections, raises important considerations for patient safety and infection control.

The finding of pathogenic germs on sterile fields during total knee and hip arthroplasty surgeries highlights the potential for contamination despite strict adherence to sterile techniques. The presence of pathogens on sterile fields may be attributed to factors such as improper sterilisation techniques, inadequate adherence to aseptic practices, or the introduction of contaminants during the surgical process.

There is not a significant influence of temperature on the results of surgical interventions and the appearance of pathogens in the operating room. At 18°C, 14 additional pathogens were identified by the end of the operation, at 20°C, there were 12 more pathogens, and at 22°C, the number increased by four only at the end. The rise in pathogenic germs with increasing temperature suggests a potential link to the development of Surgical Site Infections (SSIs). Maintaining an optimal temperature and having an effective ventilation system [7] can reduce the risk of SSIs and can promote better patient outcomes [13].

Hammond et al. affirmed that significant deviations in intraoperative temperature are not associated with increased risk of SSI. Temperature variations in the operating room can also impact the immune response of both patients and surgical staff. At lower temperatures, the body's immune system may be compromised, making patients more susceptible to infections. In contrast, higher temperatures can lead to dehydration and heat stress, weakening the immune response of both patients and healthcare workers. A weakened immune system can increase the likelihood of infections during and after surgery. Vaishya et al. [25] reported that the temperature must variate between 21°C and 23°C like we reported in our study also. Nowadays, there are also standards for medical clothing, made for use in the operating theatre, that have been developed with the progress in science and technology [30]. Further information and, for sure, better trials are required to manage the impact of different operating room clothing on SSi rates [19].

Given the critical implications of temperature in the operating room, maintaining a controlled and consistent temperature becomes imperative. Regular monitoring and adjustment of the temperature during surgical procedures are essential to ensure the comfort of both patients and surgical staff and to prevent the proliferation of pathogens.

The study's findings indicate a correlation between the operating table height and the presence of pathogens during surgery. The increase in the number of pathogens observed at lower table heights (100 cm) suggests that having the table closer to the ground might increase the risk of contamination from the surrounding environment. The study showed that a table height of 100 cm led to the appearance of six new pathogens after 60 min and an additional 10 by the end of the operation. In contrast, at a table height of 150 cm, no new pathogens appeared after 60 min, but six were detected by the end of the surgery. The height of the operating table can also influence the exposure to airborne contaminants in the operating room. If the table is positioned too low, it might be more susceptible to air currents that could carry particles, including pathogens, towards the surgical site. Proper positioning of the operating table can help reduce exposure to potential airborne contaminants and improve the overall air quality in the surgical environment. Judging by the *p*‐value on operating table height we can say that this is not a significant factor that should be considered in infection control protocols. Anyway, adjustable tables allow surgeons and medical staff to customise the table's height and position according to the specific surgical procedure and individual ergonomic needs. This adaptability can lead to improved surgical performance and reduced risk of pathogen transmission.

The high percentage of germs identified on the instrument table at the beginning of the operation is a cause for concern. This highlights the importance of thorough sterilisation and disinfection procedures before the surgery starts. The increase in the percentage of pathogens at the end of the operation and one hour into the intervention underscores the need for maintaining a sterile environment during the entire surgical procedure.

The presence of pathogens at the incision site raises the risk of SSIs, which can significantly impact patient outcomes. 396 samples were collected during 33 interventions, with 74 testing positive for various pathogens. The most prevalent pathogen was *S. epidermidis*, accounting for 45.45% of the cases, followed by Sphingomonas paucimobilis at 18.31%, and Proteus vulgaris at 9%. Notably, *S. aureus*, a common pathogen in orthopaedic infections, was not identified in this study. The consistent percentage of pathogens on the sterile field at different distances from the incision site indicates the need for effective measures to prevent microbial migration within the surgical area. Kanamori et al. [15] reported that the most common bacteria found in operating room is *S. aureus* while, in our study, we discovered a high percentage of *S. epidermidis*.

Most germs were found on the instrument table, starting at 17.18% at the beginning of the operation and increasing to 26.27% by the end, with an intermediate 8% at one hour. At the incision site, pathogens were 8% initially and at one hour, rising to 17.18% by the operation's end. Pathogen levels on the sterile field remained constant at 8% and 3.54% at 10 and 50 cm from the incision, respectively, throughout the procedure.

The absence of identified pathogens on the handles of the lighting lamps is a positive finding, suggesting that proper cleaning and maintenance protocols are effective in preventing contamination in this area. However, it is important not to overlook potential areas of cross‐contamination and continue to prioritise infection control practices throughout the operating room.

The correlation between the number of door openings and the appearance of new pathogens underscores the importance of maintaining a controlled and restricted access environment in the operating room. Mears et al. [20] reported a frequency of 16.6–37.3 door openings in one hour during the joint replacement surgery while in our study we have reported between 45 and 60 door openings. Each door opening creates the potential for introducing contaminants from the surrounding areas, necessitating careful monitoring and minimising unnecessary door movements during surgical procedures.

The inverse relationship between the number of touches and the appearance of new pathogens on sterile fields highlights the significance of reducing unnecessary contact with sterile surfaces.

The link between the number of people in the operating room and the appearance of new pathogens emphasises the importance of managing the number of personnel present during surgery. Cristina et al. [4] reported a safe number of 5 ± 1 persons in OR while we consider that more than seven persons being there during the surgery can increase the risk of infections. Overcrowding can lead to increased microbial dispersion, underscoring the need for optimising personnel and maintaining a well‐organised and controlled surgical environment.

The findings from this study emphasise the critical role of infection control practices in the operating room. Adhering to strict protocols for sterilisation, disinfection, aseptic techniques, and limiting unnecessary movement can significantly reduce the risk of pathogen transmission and SSIs. An incidence of SSIs of 0.3%–2.5% after hip o knee arthroplasty has been reported [28] while in our study there is no site infection.

Prevention should be mandatory and antibiotics should be recognised as a cornerstone [24]. Prophylactic administration of antibiotics plays a critical role in reducing the risk of periprosthetic infections. However, it is essential to strike a balance between effective prophylaxis and minimising the development of antibiotic resistance. Careful consideration should be given to selecting appropriate antibiotics based on the antibiogram data and established guidelines.

In the context of periprosthetic infections, a comprehensive approach is necessary. This includes optimising surgical techniques, implementing rigorous infection control measures, proper sterilisation protocols, appropriate wound care, and effective antimicrobial prophylaxis. Additionally, the findings from the study can contribute to ongoing research and the development of strategies to further reduce the incidence of periprosthetic infections.

The patients postoperative progress was favourable at 3 and 6 months, with no discharge at the wound site and no signs of inflammation and normal complete blood count (CBC) and normal inflammatory markers.

Limitations to consider in this study include the small number of samples, the specific context of the SUCH, potential confounding factors and very important, the absence of negative controls. Further studies with larger sample sizes, multi‐centre collaboration, and long‐term follow‐up can provide more robust evidence and help refine strategies for preventing periprosthetic infections.

## CONCLUSIONS

Maintaining a specific temperature and the height of the operating table in the operating room is not significant for OR contamination. A higher number of individuals in the OR, lamp touches and door opening significantly contributes to contamination. *S. epidermidis* is the most commonly encountered bacterium in reported interventions.

## AUTHOR CONTRIBUTIONS

**Nicolas C. I. Ion**: Conceptualisation; methodology; data curation; writing—original draft preparation. **Sorin R. Fleaca**: Conceptualisation; validation; data curation. **Bogdan‐Axente Bocea**: Writing—original draft preparation. **Cosmin‐Ioan Mohor**: Validation. **Mihai‐Dan Roman**: Validation. **Alexandru‐Florin Diconi**: Methodology. **Adrian N. Cristian**: Methodology. **Doru‐Florian‐Cornel Moga**: Data curation. **Mona Y. Cataniciu**: Data curation. **Ovidiu N. Popa**: Writing—original draft preparation. **Adrian G. Boicean**: Methodology. **Victoria Birlutiu**: Validation; supervision. All authors have read and agreed to the published version of the manuscript.

## CONFLICT OF INTEREST STATEMENT

The authors declare no conflicts of interest.

## ETHICS STATEMENT

This article does not contain any studies with human participants or animals performed by any of the authors.

## Data Availability

The datasets used and/or analysed during the current study are available from the corresponding author on reasonable request.
